# Synthesis, Structures, and Photophysical Properties of Novel Four-Coordinate Cu(I) Complexes Supported by Chelating N-Heterocyclic Carbene Ligands

**DOI:** 10.3389/fchem.2019.00422

**Published:** 2019-06-05

**Authors:** Zhiqiang Wang, Xiaojuan Sun, Chen Xu, Baoming Ji

**Affiliations:** ^1^College of Chemistry and Chemical Engineering and Henan Key Laboratory of Function-Oriented Porous Materials, Luoyang Normal University, Luoyang, China; ^2^College of Food and Pharmacy, Luoyang Normal University, Luoyang, China

**Keywords:** Cu(I) complexes, N-heterocyclic carbene, thermally activated delayed fluorescence, crystal structures, theoretical calculations

## Abstract

Luminescent Cu(I) complexes are promising emitting materials for electroluminescent devices due to their low cost and abundant resources, as well as high emission efficiency. It is well-known that N-heterocyclic carbenes (NHCs) are excellent ligands for transition metal complexes. To investigate the photophysical properties of Cu(I)-NHC complexes, a series of new mononuclear four-coordinate Cu(I) complexes supported by the diphosphine ligand bis[2-(diphenylphosphino)phenyl]ether (POP) and the NHC ligands, consisting of imidazolylidene and pyrimidine units, were synthesized and fully characterized. To tune the photophysical properties of these Cu(I)-NHC complexes, the NHC ligands were attached with electron-withdrawing/donating groups (fluorine, chlorine, methyl and methoxyl) at the pyrimidine unit. All of these Cu(I)-NHC complexes adopt the typical distorted tetrahedral configuration. The electron-donating groups can lead to shorter Cu–N bond distances and longer Cu–C bond distances compared to the electron-withdrawing groups. Theoretical calculation results show that the highest occupied molecular orbitals are mainly distributed on the Cu(I) ion, POP, and carbene unit, while the lowest unoccupied molecular orbitals are mostly located on the pyrimidine unit of NHC ligands. The lowest energy electronic transitions of these Cu(I)-NHC complexes are mainly the metal-to-ligand charge transfer transition and ligand-to-ligand charge transfer transition. These Cu(I)-NHC complexes in solid state show tunable emissions from 530 to 618 nm with efficiencies of 0.5–38.1% at room temperature. The photophysical behaviors of these complexes at 298 and 50 K match well with the thermally activated delayed fluorescence (TADF) characteristics.

## Introduction

Since the efficient organic-light emitting diodes (OLEDs) and light-emitting electrochemical cells (LEECs) based on Cu(I) complexes were reported by Wang group and Armaroli group (Zhang et al., 2004; Armaroli et al., 2006), respectively, luminescent Cu(I) complexes have been attracting considerable attention as the emitting materials for electroluminescent (EL) devices (Volz et al., 2014; Gneuß et al., 2015; Hofbeck et al., 2015; Kobayashi et al., 2016; Brunner et al., 2017; He et al., 2017; Huang et al., 2017; Su et al., 2017; Brown et al., 2018; Mohankumar et al., 2018; Schinabeck et al., 2018; Jia et al., 2019). According to the recent reports, most of Cu(I) complexes show thermally activated delayed fluorescence (TADF) due to small energy gaps (Δ*E*_ST_) between the lowest singlet state (S_1_) and the lowest triplet states (T_1_) (Gneuß et al., 2015; Hofbeck et al., 2015; Kobayashi et al., 2016; Brunner et al., 2017; Huang et al., 2017; Su et al., 2017; Mohankumar et al., 2018; Schinabeck et al., 2018; Jia et al., 2019). As we all know, singlet and triplet excitons are formed in a ratio of 1:3 during EL device operation. In the EL devices based on TADF materials, T_1_ excitons can be translated to S_1_ excitons through reverse intersystem crossing (RISC), and all S_1_ excitons will be converted into photons by the spin-allowed S_1_ → S_0_ transition (Wang K. et al., 2017; Shi et al., 2018). Thus, the EL devices using TADF Cu(I) complexes as emitters can utilize both singlet and triplet excitons to generate photons resulting in high EL efficiencies. For this reason, TADF Cu(I) complexes are seen as promising alternatives to the phosphorescent complexes based on noble metals, such as Ir(III) and Pt(II) complexes.

Photophysical properties of the homoleptic Cu(I) complexes supported by diimine ligands (general formula [Cu(N^∧^N)_2_]^+^) have always been the focus of research for a long time (Simon et al., 1996; Felder et al., 2001; Kovalevsky et al., 2003; Kalsani and Schmittel, 2006; Leydet et al., 2007). Although many significant research results were obtained, these Cu(I) complexes only show very low emission efficiencies. In 2002, McMillin group used a chelating diphosphine ligand to prepare heteroleptic Cu(I) complexes first, which brought a great breakthrough in the emission efficiency of luminescent Cu(I) complexes (Cuttell et al., 2002; Kuang et al., 2002). For example, the photoluminescence quantum yield (ϕ_PL_) of [Cu(dbp)(POP)]^+^ is hundreds-fold larger than those of [Cu(N^∧^N)_2_]^+^, where POP = bis[2-(diphenylphosphino)phenyl]ether, dbp = 2,9-di-*n*-butyl-1,10-phenanthroline. In recent years, lots of luminescent heteroleptic Cu(I) complexes supported by chelating diphosphine ligands and diimine ligands (general formula [Cu(P^∧^P)(N^∧^N)]^+^) have been successfully developed, and efficient EL devices based on this kind of Cu(I) complexes have been fabricated (Cheng et al., 2015; Osawa et al., 2015; Liang et al., 2016; Lin et al., 2017; Zhang et al., 2017; Alkan-Zambada et al., 2018; Keller et al., 2018; Liu et al., 2018; Brunner et al., 2019).

**Scheme 1:**
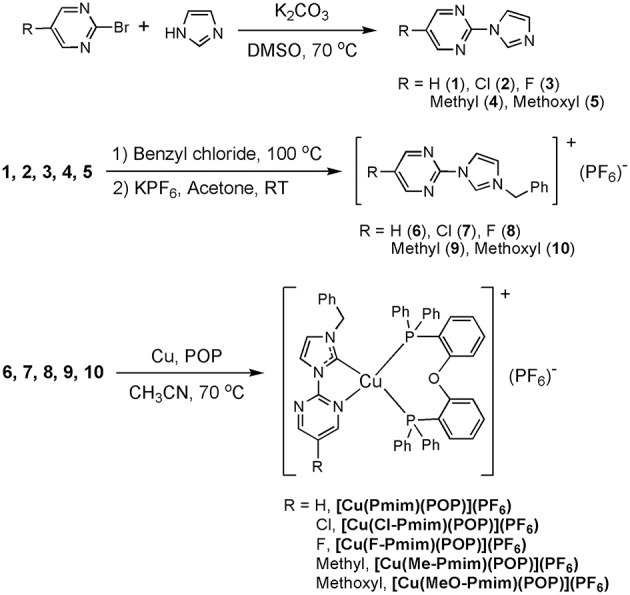
Synthesis routes of the Cu(I)-NHC complexes.

It is well known that N-heterocyclic carbenes (NHCs) are excellent ligands for transition metal complexes because of their strong σ-donating ability and modest π-accepting ability. Moreover, it has been proven that two- and three-coordinate Cu(I) complexes supported by NHC ligands can give efficient TADF or phosphorescence (Krylova et al., 2012; Leitl et al., 2014; Marion et al., 2014; Elie et al., 2016; Nishikawa et al., 2016; Hamze et al., 2017; Lu et al., 2018). In view of these findings, we synthesized several four-coordinate Cu(I)-NHC complexes with efficient TADF by replacing the diimine ligands in Cu(P^∧^P)(N^∧^N)]^+^ with the chelating NHC ligands consisting of imidazolylidene and pyridine (Wang et al., 2016, 2018), and the Zhao group also carried out similar research (Liu et al., 2017; Wang J. et al., 2017; Xu et al., 2018). In this paper, we designed a series of new chelating NHC ligands, in which the imidazolylidene unit is connected with the pyrimidine unit (Scheme 1). Five luminescent Cu(I)-NHC complexes supported by these NHC ligands and the diphosphine ligand POP were successfully synthesized and fully characterized. Research results indicate that the electron-withdrawing/donating groups at pyrimidine unit can effectively tune the photophysical properties of these Cu(I)-NHC complexes, and the emission behaviors of these complexes at 50 K and 298 K match well with TADF characters.

## Experimental

### General Methods

^1^H NMR, ^13^C NMR, and ^31^P NMR spectra were recorded on the Bruker Avance 400 spectrometer and Bruker Avance 500 spectrometer. ^1^H and ^13^C chemical shifts are referenced to the internal tetramethylsilane (δ = 0 ppm) and ^31^P chemical shifts were referenced to the external 85% phosphoric acid (δ = 0 ppm). Mass spectra (MS) were obtained on a Bruker Bruker APEX II FT-ICR instrument. Elemental analysis was performed on a Vario III elemental analyzer. UV–visible absorption spectra were recorded on a Hitachi U-3010 UV–vis spectrophotometer. Photoluminescence (PL) spectra, emission lifetimes, and absolute emission quantum yields were recorded on an Edinburgh FLS980 spectrometer equipped with an integrating sphere.

### Synthesis

All starting materials were purchased from commercial suppliers and used as received. Solvents are analytical grade and used without further purification unless otherwise stated.

#### 2-(1H-imidazol-1-yl)pyrimidine (1)

2-bromopyrimidine (790 mg, 5 mmol), imidazole (680 mg, 5 mmol), K_2_CO_3_ (1.39 g, 10 mmol) and DMSO (15 mL) were added in a round-bottomed flask, then the reaction mixture was stirred vigorously for 24 h at 70°C. After cooling, ethyl acetate (30 mL) was added to the mixture, and then the organic solution was washed with water, dried over anhydrous MgSO_4_ and evaporated with a rotary evaporator. The residue was purified by column chromatography using ethyl acetate/dichloromethane (1:1 v/v) as a eluent to give the desired product as white powder. Yield: 569 mg (78%). ^1^H NMR (400 MHz, CDCl_3_) δ (ppm): 8.70 (2H, d, *J* = 4.8), 8.63 (1H, s), 7.90 (1H, s), 7.21 (1H, t, *J* = 4.8), 7.18 (1H, s). MS (*m*/*z*, ESI): 147.1 [M+H]^+^.

#### 5-chloro-2-(1H-imidazol-1-yl)pyrimidine (2)

2-bromo-5-chloropyrimidine (965 mg, 5 mmol), imidazole (680 mg, 5 mmol), K_2_CO_3_ (1.39 g, 10 mmol) and DMSO (15 mL) were added in a round-bottomed flask, then the reaction mixture was stirred vigorously for 24 h at 70°C. After cooling, ethyl acetate (30 mL) was added to the mixture, and then the organic solution was washed with water, dried over anhydrous MgSO_4_ and evaporated with a rotary evaporator. The residue was purified by column chromatography using ethyl acetate/dichloromethane (1:1 v/v) as eluent to give the desired product as white powder. Yield: 730 mg (81%). ^1^H NMR (400 MHz, CDCl_3_) δ (ppm): 8.57 (2H, s), 8.50 (1H, s), 7.77 (1H, s), 7.10 (1H, s). MS (*m*/*z*, ESI): 181.0 [M+H]^+^.

#### 5-fluoro-2-(1H-imidazol-1-yl)pyrimidine (3)

2-bromo-5-fluoropyrimidine (885 mg, 5 mmol), imidazole (680 mg, 5 mmol), K_2_CO_3_ (1.39 g, 10 mmol) and DMSO (15 mL) were added in a round-bottomed flask, then the reaction mixture was stirred vigorously for 24 h at 70°C. After cooling, ethyl acetate (30 mL) was added to the mixture, and then the organic solution was washed with water, dried over anhydrous MgSO_4_, and evaporated with a rotary evaporator. The residue was purified by column chromatography using ethyl acetate/dichloromethane (1:1 v/v) as eluent to give the desired product as white powder. Yield: 697 mg (85%). ^1^H NMR (400 MHz, CDCl_3_) δ (ppm): 8.57 (2H, s), 8.55 (1H, s), 7.83 (1H, d, *J* = 0.8), 7.17 (1H, d, *J* = 0.4). MS (*m*/*z*, ESI): 165.1 [M+H]^+^.

#### 2-(1H-imidazol-1-yl)-5-methylpyrimidine (4)

2-bromo-5-methylpyrimidine (865 mg, 5 mmol), imidazole (680 mg, 5 mmol), K_2_CO_3_ (1.39 g, 10 mmol) and DMSO (15 mL) were added in a round-bottomed flask, then the reaction mixture was stirred vigorously for 24 h at 70°C. After cooling, ethyl acetate (30 mL) was added to the mixture, and then the organic solution was washed with water, dried over anhydrous MgSO_4_ and evaporated with a rotary evaporator. The residue was purified by column chromatography using ethyl acetate/dichloromethane (1:1 v/v) as eluent to give the desired product as white powder. Yield: 576 mg (72%). ^1^H NMR (400 MHz, CDCl_3_) δ (ppm): 8.58 (1H, s), 8.49 (2H, s), 7.86 (1H, s), 7.15 (1H, s), 2.33 (3H, s). ^13^C NMR (100 MHz, CDCl_3_) δ (ppm): 158.66, 153.18, 136.09, 130.58, 128.40, 116.55, 15.21. HRMS (*m*/*z*, ESI): 161.0823 [M+H]^+^.

#### 2-(1H-imidazol-1-yl)-5-methoxypyrimidine (5)

2-bromo-5-methoxypyrimidine (945 mg, 5 mmol), imidazole (680 mg, 5 mmol), K_2_CO_3_ (1.39 g, 10 mmol) and DMSO (15 mL) were added in a round-bottomed flask, then the reaction mixture was stirred vigorously for 24 h at 70°C. After cooling, ethyl acetate (30 mL) was added to the mixture, and then the organic solution was washed with water, dried over anhydrous MgSO_4_ and evaporated with a rotary evaporator. The residue was purified by column chromatography using ethyl acetate/dichloromethane (1:1 v/v) as eluent to give the desired product as white powder. Yield: 616 mg (70%). ^1^H NMR (400 MHz, CDCl_3_) δ (ppm): 8.52 (1H, s), 8.34 (2H, s), 7.82 (1H, s), 7.15 (1H, s), 3.95 (3H, s). ^13^C NMR (100 MHz, CDCl_3_) δ (ppm): 151.97, 148.79, 144.63, 135.88, 130.46, 116.60, 56.47. HRMS (*m*/*z*, ESI): 177.0772 [M+H]^+^.

#### Compound 6

In a round-bottomed flask, a mixture of benzyl chloride (1.27 g, 10 mmol) 2-(1H-imidazol-1-yl)pyrimidine (584 mg, 4 mmol) was refluxed at 100°C for 2 h. After the excess benzyl chloride was evaporated under vacuum, the residue was put in acetone (20 mL). Then KPF_6_ (1.84 g, 10 mmol) was added and stirred for 3 h. The precipitate KCl was filtrated and the organic solvent was evaporated with a rotary evaporator to give the desired product as white powder. Yield: 1.31 g (85%). ^1^H NMR (400 MHz, Acetone-D6) δ (ppm): 10.16 (1H, s), 9.04 (2H, d, *J* = 4.8), 8.52 (1H, s), 7.96 (1H, s), 7.77 (1H, t, *J* = 4.8), 7.64–7.62 (2H, m), 7.49–7.41 (3H, m), 5.78 (2H, s). ^13^C NMR (100 MHz, Acetone-D6) δ (ppm): 160.86, 153.42, 136.52, 134.61, 130.17, 130.13, 129.72, 124.72, 123.49, 120.75, 54.57. Anal. Calcd. for C_14_H_13_F_6_N_4_P: C 43.99, H 3.43, N 14.66; Found: C 44.07, H 3.46, N 14.69.

#### Compound 7

In a round-bottomed flask, a mixture of benzyl chloride (1.27 g, 10 mmol) 5-chloro-2-(1H-imidazol-1-yl)pyrimidine (720 mg, 4 mmol) was refluxed at 100°C for 2 h. After the excess benzyl chloride was evaporated under vacuum, the residue was put in acetone (20 mL). Then KPF_6_ (1.84 g, 10 mmol) was added and stirred for 3 h. The precipitate KCl was filtrated and the organic solvent was evaporated with a rotary evaporator to give the desired product as white powder. Yield: 1.38 g (83%). ^1^H NMR (400 MHz, Acetone-D6) δ (ppm): 10.17 (1H, s), 9.11 (2H, s), 8.50 (1H, s), 7.99 (1H, s), 7.63–7.61 (2H, m), 7.48–7.45 (3H, m), 5.79 (2H, s). ^13^C NMR (100 MHz, Acetone-D6) δ (ppm): 159.20, 151.60, 136.81, 134.55, 132.51, 130.23, 130.16, 129.75, 124.92, 120.99, 54.67. Anal. Calcd. for C_14_H_12_ClF_6_N_4_P: C 40.35, H 2.90, N 13.45; Found: C 40.42, H 2.88, N 13.48.

#### Compound 8

In a round-bottomed flask, a mixture of benzyl chloride (1.27 g, 10 mmol) 5-fluoro-2-(1H-imidazol-1-yl)pyrimidine (656 mg, 4 mmol) was refluxed at 100°C for 2 h. After the excess benzyl chloride was evaporated under vacuum, the residue was put in acetone (20 mL). Then KPF_6_ (1.84 g, 10 mmol) was added and stirred for 3 h. The precipitate KCl was filtrated and the organic solvent was evaporated with a rotary evaporator to give the desired product as white powder. Yield: 1.30 g (81%). ^1^H NMR (400 MHz, Acetone-D6) δ (ppm): 10.14 (1H, s), 9.06 (2H, s), 8.50 (1H, s), 7.98 (1H, s), 7.62–7.56 (2H, m), 7.47–7.43 (3H, m), 5.79 (2H, s). ^13^C NMR (100 MHz, Acetone-D6) δ (ppm): 159.00 (*J* = 261.4), 149.27, 148.72 (*J* = 24), 136.68, 134.60, 130.21, 130.16, 129.73, 124.85, 121.07, 54.61. Anal. Calcd. for C_14_H_12_F_7_N_4_P: C 42.01, H 3.02, N 14.00; Found: C 42.08, H 3.04, N 14.03.

#### Compound 9

In a round-bottomed flask, a mixture of benzyl chloride (1.27 g, 10 mmol) 2-(1H-imidazol-1-yl)-5-methylpyrimidine (640 mg, 4 mmol) was refluxed at 100°C for 2 h. After the excess benzyl chloride was evaporated under vacuum, the residue was put in acetone (20 mL). Then KPF_6_ (1.84 g, 10 mmol) was added and stirred for 3 h. The precipitate KCl was filtrated and the organic solvent was evaporated with a rotary evaporator to give the desired product as white powder. Yield: 1.39 g (88%). ^1^H NMR (400 MHz, Acetone-D6) δ (ppm): 10.10 (1H, s), 8.87 (2H, s), 8.47 (1H, s), 7.94 (1H, s), 7.64–7.61 (2H, m), 7.47–7.41 (3H, m), 5.77 (2H, s), 2.46 (3H, s). ^13^C NMR (100 MHz, Acetone-D6) δ (ppm): 160.48, 151.60, 136.22, 134.66, 133.81, 130.16, 130.13, 129.73, 124.61, 120.65, 54.53, 15.14. Anal. Calcd. for C_15_H_15_F_6_N_4_P: C 45.46, H 3.82, N 14.14; Found: C 43.35, H 3.79, N 14.11.

#### Compound 10

In a round-bottomed flask, a mixture of benzyl chloride (1.27 g, 10 mmol) 2-(1H-imidazol-1-yl)-5-methoxypyrimidine (704 mg, 4 mmol) was refluxed at 100°C for 2 h. After the excess benzyl chloride was evaporated under vacuum, the residue was put in acetone (20 mL). Then KPF_6_ (1.84 g, 10 mmol) was added and stirred for 3 h. The precipitate KCl was filtrated and the organic solvent was evaporated with a rotary evaporator to give the desired product as white powder. Yield: 1.47 g (89%). ^1^H NMR (400 MHz, Acetone-D6) δ (ppm): 10.04 (1H, s), 8.70 (2H, s), 8.43 (1H, s), 7.94 (1H, s), 7.63–7.61 (2H, m), 7.48–7.43 (3H, m), 5.76 (2H, s), 4.09 (3H, s). ^13^C NMR (100 MHz, Acetone-D6) δ (ppm): 155.41, 146.59, 146.32, 135.84, 134.73, 130.13, 129.71, 124.56, 120.68, 57.53, 54.46. Anal. Calcd. for C_15_H_15_F_6_N_4_OP: C 43.70, H 3.67, N 13.59; Found: C 43.61, H 3.63, N 13.56.

#### [Cu(Pmim)(POP)](PF_6_)

Under N_2_ atmosphere, compound 6 (382 mg, 1 mmol), copper powder (77 mg, 1.2 mmol) and POP (538 mg, 1 mmol) reacted in CH_3_CN (10 mL) at 70 °C overnight. After cooling, the resulting mixture was filtered, then the filtrate was collected and evaporated under vacuum. The residue was dissolved in dichloromethane/ethanol solution, and product was obtained as a pale-yellow crystal by slowly evaporating the solvent. Yield: 0.54 g (55%). ^1^H NMR (500 MHz, CD_3_CN) δ (ppm): 8.45 (2H, d, *J* = 5.0), 8.07 (1H, d, *J* = 2.0), 7.39–7.26 (7H, m), 7.32–7.13 (16H, m), 7.07–6.96 (8H, m), 6.80 (2H, d, *J* = 7.5), 6.68–6.64 (2H, m), 5.14 (2H, s); ^13^C NMR (125 MHz, CD_3_CN) δ (ppm):158.75, 158.18, 158.14, 158.09, 155.33, 135.72, 134.19, 133.50, 132.86, 132.20, 130.23, 129.99, 128.81, 128.77, 127.99, 125.10, 123.89, 123.77, 123.66, 122.85, 120.52, 119.75, 117.96, 54.94; ^31^P NMR (202 MHz, CD_3_CN) δ (ppm): −9.75 (s), −144.62 (quint). Anal. Calcd. for C_50_H_40_CuF_6_N_4_OP_3_: C 61.07, H 4.10, N 5.70; Found: C 61.19, H 4.15, N 5.68.

#### [Cu(Cl-Pmim)(POP)](PF_6_)

Under N_2_ atmosphere, compound 7 (416 mg, 1 mmol), copper powder (77 mg, 1.2 mmol) and POP (538 mg, 1 mmol) reacted in CH_3_CN (10 mL) at 70°C overnight. After cooling, the resulting mixture was filtered, then the filtrate was collected and evaporated under vacuum. The residue was dissolved in dichloromethane/ethanol solution, and product was obtained as a yellow crystal by slowly evaporating the solvent. Yield: 0.64 g (63%). ^1^H NMR (500 MHz, CD_3_CN) δ (ppm): 8.31 (2H, s), 8.03 (1H, d, *J* = 2.0), 7.51–7.35 (7H, m), 7.33–7.15 (14H, m), 7.13 (1H, d, *J* = 2.0), 7.09–6.92 (8H, m), 6.83 (2H, d, *J* = 7.5), 6.69–6.63 (2H, m), 5.19 (2H, s); ^13^C NMR (125 MHz, CD_3_CN) δ (ppm):164.33, 158.02, 157.97, 157.92, 156.73, 153.40, 135.54, 134.22, 133.77, 132.73, 132.32, 130.45, 130.00, 128.80, 128.44, 128.07, 125.23, 123.63, 123.51, 123.08, 120.34, 118.21, 55.10; ^31^P NMR (202 MHz, CD_3_CN) δ (ppm):−9.53 (s),−144.63 (quint). Anal. Calcd. for C_50_H_39_ClCuF_6_N_4_OP_3_: C 59.00, H 3.86, N 5.50; Found: C 59.13, H 3.82, N 5.47.

#### [Cu(F-Pmim)(POP)](PF_6_)

Under N_2_ atmosphere, compound 8 (400 mg, 1 mmol), copper powder (77 mg, 1.2 mmol) and POP (538 mg, 1 mmol) reacted in CH_3_CN (10 mL) at 70°C overnight. After cooling, the resulting mixture was filtered, then the filtrate was collected and evaporated under vacuum. The residue was dissolved in dichloromethane/ethanol solution, and product was obtained as a yellow crystal by slowly evaporating the solvent. Yield: 0.59 g (59%). Yield: 59%. ^1^H NMR (500 MHz, CD_3_CN) δ (ppm): 8.29 (2H, s), 8.03 (1H, d, *J* = 2.0), 7.49–7.35 (7H, m), 7.33–7.27 (14H, m), 7.13 (1H, d, *J* = 2.0), 7.08–6.95 (8H, m), 6.82 (2H, d, *J* = 7.5), 6.69–6.64 (2H, m), 5.17 (2 H, s); ^13^C NMR (125 MHz, CD_3_CN) δ (ppm): 158.06, 158.01, 157.96, 156.12 (*J* = 258.5), 151.52, 146.28 (*J* = 24), 135.61, 134.22, 133.66, 132.82, 132.31, 130.36, 130.03, 128.85, 128.05, 127.55, 125.22, 123.62, 123.51, 123.39, 120.41, 118.29, 54.99; ^31^P NMR (202 MHz, CD_3_CN) δ (ppm): −9.79 (s), −144.64 (quint). Anal. Calcd. for C_50_H_39_CuF_7_N_4_OP_3_: C 59.97, H 3.93, N 5.60; Found: C 59.87, H 3.89, N 5.58.

#### [Cu(Me-Pmim)(POP)](PF_6_)

Under N_2_ atmosphere, compound 9 (396 mg, 1 mmol), copper powder (77 mg, 1.2 mmol) and POP (538 mg, 1 mmol) reacted in CH_3_CN (10 mL) at 70°C overnight. After cooling, the resulting mixture was filtered, then the filtrate was collected and evaporated under vacuum. The residue was dissolved in dichloromethane/ethanol solution, and product was obtained as a colorless crystal by slowly evaporating the solvent. Yield: 0.66 g (66%). ^1^H NMR (500 MHz, CD_3_CN) δ (ppm): 8.20 (2H, s), 8.04 (1H, d, *J* = 2.0), 7.48–7.34 (7H, m), 7.32–7.26 (14H, m), 7.11 (1H, d, *J* = 2.5), 7.08–6.94 (8H, m), 6.80 (2H, d, *J* = 7.5), 6.69–6.65 (2H, m), 5.16 (2 H, s), 2.19 (3H, s); ^13^C NMR (125 MHz, CD_3_CN) δ (ppm): 158.26, 158.15, 158.10, 158.06, 153.55, 135.78, 134.16, 133.65, 132.77, 132.20, 130.30, 129.87, 129.69, 127.98, 127.51, 125.11, 123.90, 123.79, 122.62, 120.39, 117.86, 54.94, 14.09; ^31^P NMR (202 MHz, CD_3_CN) δ (ppm): −9.43 (s), −144.63 (quint). Anal. Calcd. for C_51_H_42_CuF_6_N_4_OP_3_: C 61.42, H 4.24, N 5.62; Found: C 61.31, H 4.20, N 5.65.

#### [Cu(MeO-Pmim)(POP)](PF_6_)

Under N_2_ atmosphere, compound 10 (412 mg, 1 mmol), copper powder (77 mg, 1.2 mmol) and POP (538 mg, 1 mmol) reacted in CH_3_CN (10 mL) at 70°C overnight. After cooling, the resulting mixture was filtered, then the filtrate was collected and evaporated under vacuum. The residue was dissolved in dichloromethane/ethanol solution, and product was obtained as a greenish crystal by slowly evaporating the solvent. Yield: 0.65 g (64%). ^1^H NMR (500 MHz, CD_3_CN) δ (ppm): 8.00 (1H, d, *J* = 2.0), 7.99 (2H, s), 7.50–7.38 (7H, m), 7.36–7.28 (14H, m), 7.10 (1H, d, *J* = 2.0), 7.08–6.89 (8H, m), 6.83 (2H, d, *J* = 7.5), 6.65–6.62 (2H, m), 5.25 (2H, s), 3.56 (3H, s); ^13^C NMR (125 MHz, CD_3_CN) δ (ppm): 158.06, 157.98, 157.93, 152.25, 135.88, 134.17, 133.60, 132.67, 132.25, 130.26, 128.89, 128.71, 127.97, 127.54, 125.21, 123.82, 123.70, 122.51, 120.32, 117.83, 56.28, 55.03; ^31^P NMR (202 MHz, CD_3_CN) δ (ppm): −9.44 (s), −144.65 (quint). Anal. Calcd. for C_51_H_42_CuF_6_N_4_O_2_P_3_: C 60.45, H 4.18, N 5.53; Found: C 60.33, H 4.12, N 5.56.

### X-Ray Crystallographic Analysis

Diffraction data for these complexes were collected on a Bruker SMART APEX-II CCD diffractometer with Mo Kα radiation (λ = 0.71 073 Å). The data were corrected for Lorentz polarization factors as well as for absorption. Structures were solved by direct methods and refined by full-matrix least-squares methods on *F*^2^ with the SHELXL-97 program. All non-hydrogen atoms were refined anisotropically, while H atoms were placed in geometrically calculated positions. CCDC reference numbers for [Cu(Pmim)(POP)](PF_6_), [Cu(Cl-Pmim)(POP)](PF_6_), [Cu(F-Pmim)(POP)](PF_6_), [Cu(Me-Pmim)(POP)](PF_6_) and [Cu(MeO-Pmim)(POP)](PF_6_) are 1852674, 1852675, 1852676, 1852677 and 1852678, respectively.

### Theoretical Calculations

Density functional theory (DFT) calculations were performed with B3LYP functional using Gaussian 09 program. The 6-31G^*^ basis set was used for C, N, H, O, F, Cl and P, and the LanL2DZ was used for Cu. Geometric parameters obtained from X-ray analyses were used as a starting point for geometry optimization in the ground state, and frequency calculations were performed to confirm the optimized structures to be true minima on the potential energy surfaces. Time-dependent density functional (TD-DFT) calculations used the optimized geometries. The hole and electron distributions were analyzed by using the multiwfn 3.5 program.

## Results and Discussion

### Synthesis and Structures

**Scheme 1** shows the synthesis routes of these Cu(I)-NHC complexes. The N-arylated imidazoles (**1**, **2**, **3**, **4**, **5**) and the imidazolium salts (**6**, **7**, **8**, **9**, **10**) were synthesized through the reported method (Wang et al., 2015, 2016). The target Cu(I)-NHC complexes were synthesized by the simple one-pot method reported by Zhao group (Liu et al., 2017; Wang J. et al., 2017; Xu et al., 2018). All of these complexes are very stable to air and moisture in a solid state at room temperature. The structures of these complexes were characterized by ^1^H NMR, ^13^C NMR, and ^31^P NMR, and the characteristic signal peaks from methylene, methyl, methoxyl groups in the NHC ligands and phosphorus atoms in the ligand POP and hexafluorophosphate anion can be observed clearly in the NMR spectra. Furtherly, the structures of these complexes were confirmed by X-ray crystallography.

**Figure 1:**
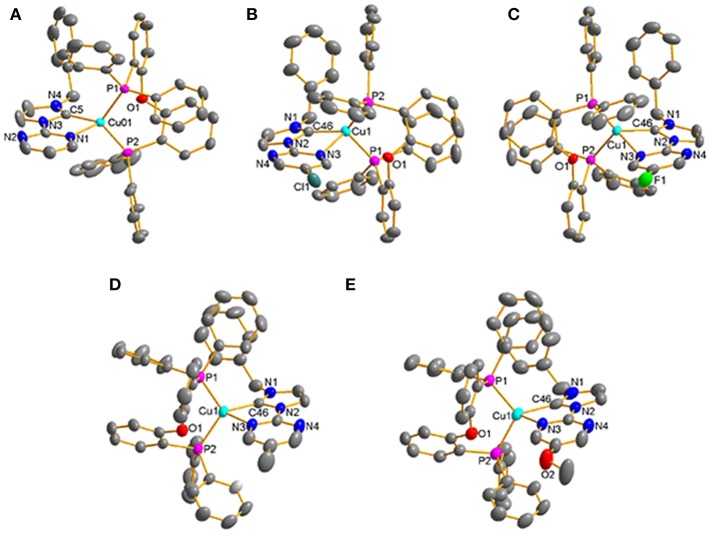
Crystal structures of the Cu(I)-NHC complexes **(A)** [Cu(Pmim)(POP)](PF_6_), **(B)** [Cu(Cl-Pmim)(POP)](PF_6_), **(C)** [Cu(F-Pmim)(POP)](PF_6_), **(D)** [Cu(Me-Pmim)(POP)](PF_6_), **(E)** [Cu(MeO-Pmim)(POP)](PF_6_) (30% probability ellipsoids, H atoms, solvent molecules and ${\text{PF}}_{6}^{-}$ ion are omitted).

All of these complexes adopt the distorted tetrahedral configuration (Figure 1), the bond lengths and bond angles around central Cu(I) ions are listed in Table 1. It can be found that the Cu–N bond lengths are in agreement with the electron-effect of substituents at the pyrimidine unit of NHC ligands. The electron-donating methyl and methoxyl groups lead to shorter Cu–N bond lengths, while the electron-withdrawing chlorine and fluorine groups lead to longer Cu–N bond lengths, 2.216(4) Å for [Cu(MeO-Pmim)(POP)](PF_6_) < 2.228(3) Å for [Cu(Me-Pmim)(POP)](PF_6_) < 2.229(2) Å for [Cu(Pmim)(POP)](PF_6_) < 2.367(2) Å for [Cu(Cl-Pmim)(POP)](PF_6_) < 2.438(2) Å for [Cu(F-Pmim)(POP)](PF_6_). It is interesting that the Cu–C bond lengths show an opposite trend to Cu–N bond lengths. The average Cu–P bond lengths fall in the range 2.2505 −2.2712 Å, which are similar to those of reported four-coordinate Cu(I)complexes (Kuang et al., 2002; Gneuß et al., 2015; Liang et al., 2016; Brunner et al., 2017, 2019; Huang et al., 2017; Zhang et al., 2017; Alkan-Zambada et al., 2018; Keller et al., 2018). The dihedral angles between the imidazolylidene rings and pyrimidine rings in NHC ligands are 5.5(5)^o^, 15.5(4)^o^, 17.3(3)^o^, 5.1(4)^o^, and 9.2(6)^o^ for [Cu(Pmim)(POP)](PF_6_), [Cu(Cl-Pmim)(POP)](PF_6_), [Cu(F-Pmim)(POP)](PF_6_), [Cu(Me-Pmim)(POP)](PF_6_) and [Cu(MeO-Pmim)(POP)](PF_6_), respectively.

**Table 1:** Selected Bond Lengths (Å) and Angles (°) of the Cu(I)-NHC complexes.

**Compounds**	**Bond lengths** **(Å)**		**Bond angles (**^****°****^**)**	
[Cu(Pmim)(POP)](PF_6_)	Cu01–C5	1.968(3)	C5–Cu01–N1	79.10(13)
	Cu01–N1	2.229(2)	C5–Cu01–P1	120.97(9)
	Cu01–P1	2.2470(9)	C5–Cu01–P2	115.61(10)
	Cu01–P2	2.2540(9)	P1–Cu01–P2	110.64(3)
			N1–Cu01–P1	107.92(7)
			N1–Cu01–P2	119.39(7)
[Cu(Cl-Pmim)(POP)](PF_6_)	Cu1–C46	1.957(3)	C46–Cu1–N3	76.55(10)
	Cu1–N3	2.367(2)	C46–Cu1–P1	113.50(9)
	Cu1–P1	2.2660(8)	C46–Cu1–P2	133.30(9)
	Cu1–P2	2.2505(8)	P1–Cu1–P2	112.22(3)
			N3–Cu1–P1	108.23(6)
			N3–Cu1–P2	97.89(6)
[Cu(F-Pmim)(POP)](PF_6_)	Cu1–C46	1.948(2)	C46–Cu1–N3	75.74(9)
	Cu1–N3	2.438(2)	C46–Cu1–P1	132.50(8)
	Cu1–P1	2.2520(7)	C46–Cu1–P2	113.71(8)
	Cu1–P2	2.2668(7)	P1–Cu1–P2	113.23(3)
			N3–Cu1–P1	96.58(5)
			N3–Cu1–P2	107.86(6)
[Cu(Me-Pmim)(POP)](PF_6_)	Cu1–C46	1.993(3)	C46–Cu1–N3	79.26(12)
	Cu1–N3	2.228(3)	C46–Cu1–P1	118.59(9)
	Cu1–P1	2.2882(8)	C46–Cu1–P2	126.83(9)
	Cu1–P2	2.2542(9)	P1–Cu1–P2	112.75(3)
			N3–Cu1–P1	102.87(8)
			N3–Cu1–P2	102.52(8)
[Cu(MeO-Pmim)(POP)](PF_6_)	Cu1–C46	2.083(6)	C46–Cu1–N3	77.79(18)
	Cu1–N3	2.216(4)	C46–Cu1–P1	119.07(13)
	Cu1–P1	2.2735(12)	C46–Cu1–P2	125.53(14)
	Cu1–P2	2.2509(14)	P1–Cu1–P2	113.45(5)
			N3–Cu1–P1	104.64(11)
			N3–Cu1–P2	102.59(11)

### Theoretical Calculations

**Figure 2:**
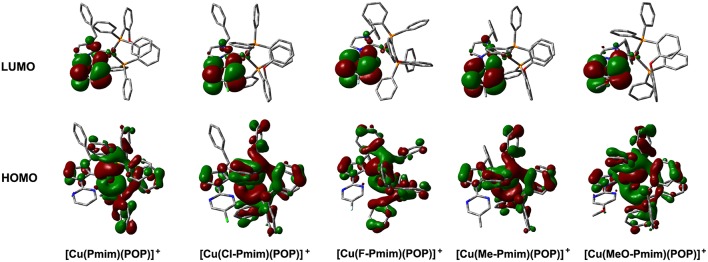
Calculated molecular orbitals for the Cu(I)-NHC complexes. The “+” denotes a positive charge. Figure shows the DFT calculation results. The DFT calculations were performed without regard to the hexafluorophosphate anion in these complexes.

To gain insight into the electronic structures of these complexes, we carried out DFT calculations using the geometric parameters obtained from X-ray analyses as a starting structure. These complexes have similar molecular orbitals (MO) as shown in Figure 2. The highest occupied molecular orbitals (HOMOs) are mainly distributed on the Cu(I) ion and the ligand POP, moreover, the imidazolylidene unit also has a certain amount of contributions to HOMOs. In contrast, the lowest unoccupied molecular orbitals (LUMOs) are mostly located on the pyrimidine unit and sparingly located on the imidazolylidene unit, while the central Cu(I) ion and the ligand POP have minimal contributions. It can be found that the overlaps between the HOMOs and LUMOs of these complexes are very small, which matches with the MO character of TADF materials (Yang et al., 2017; Wang et al., 2019). The TD-DFT calculation results show that the S_1_ and T_1_ excitations of these complexes mainly involve the HOMO → LUMO transition and HOMO → LUMO+1 transition, while the T_2_ excitations involve the lower bonding orbitals and higher anti-bonding orbitals, such as HOMO-1 → LUMO, HOMO-1 → LUMO+1, HOMO-3 → LUMO and so on (Table S1). On the basis of TD-DFT results, we analyzed the hole and electron distributions of these complexes at the S_1_ and T_1_ states. The analysis results show that the lowest energy electronic transitions of these complexes are mainly metal-to-ligand charge transfer (MLCT) transition and ligand-to-ligand charge transfer (LLCT) transition (Figure S1). These calculation results are essentially in agreement with our previous reports and similar to those of other heteroleptic four-coordinate Cu(I) complexes with TADF (Gneuß et al., 2015; Wang et al., 2016, 2018; Huang et al., 2017; Lin et al., 2017).

### Photophysical Properties

**Figure 3:**
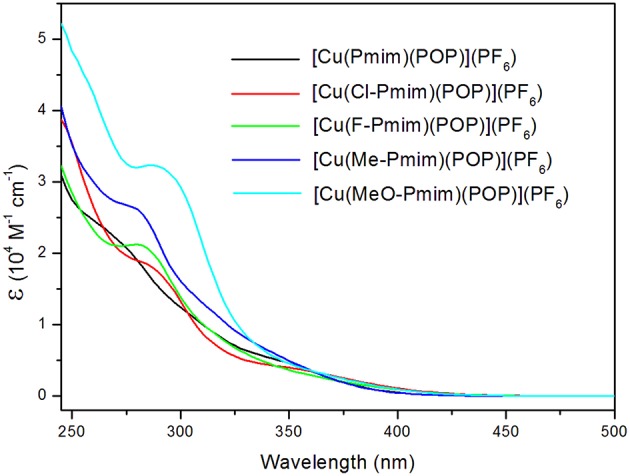
Absorption spectra of the Cu(I)-NHC complexes in CH_2_Cl_2_ solution at room temperature.

The UV-visible absorption spectra of these complexes in dichloromethane solution at room temperature are shown in Figure 3. All of these complexes exhibit intense absorption bands in the wavelength range < 330 nm, which can be assigned to the π-π^*^ transitions of ligands. By comparing with absorption spectra of the free ligand POP and the NHC ligand precursors **6**, **7**, **8**, **9** and **10** (Figure S2), the weaker absorption bands (ε < 0.85 × 10^4^ M^−1^ cm^−1^) over 330 nm of these complexes can be attributed to the charge transfer (CT) transitions, which include the MLCT and LLCT transitions according to the above calculation results. Because the emission of Cu(I) complexes can be quenched by Jahn-Teller distortion and solvent-induced exciplex (Scaltrito et al., 2000), these complexes do not show luminescence in organic solutions at room temperature.

**Figure 4:**
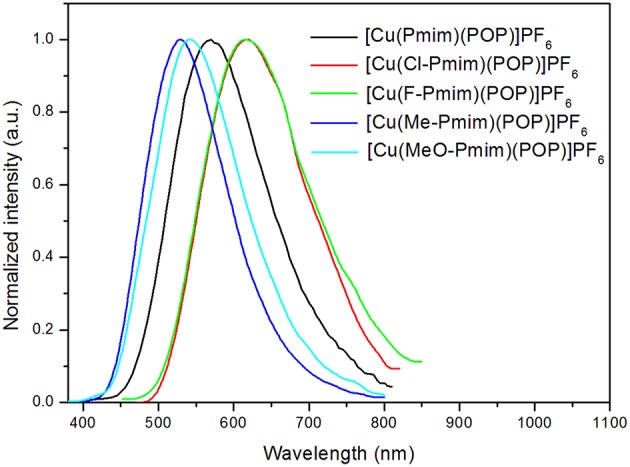
Photoluminescence spectra of the Cu(I)-NHC complexes in solid state at room temperature.

All of these complexes exhibit obvious emissions in solid state at room temperature, the emission peaks are located at 570 nm for [Cu(Pmim)(POP)](PF_6_), 618 nm for [Cu(Cl-Pmim)(POP)](PF_6_), 616 nm for [Cu(F-Pmim)(POP)](PF_6_), 530 nm for [Cu(Me-Pmim)(POP)](PF_6_) and 542 nm for [Cu(MeO-Pmim)(POP)](PF_6_), respectively (Figure 4). The photoluminescence spectra of these complexes are broad and unstructured which matches with the CT character of emissive state. It is clear that the electron-withdrawing groups (fluorine and chlorine)/electron-donating groups (methyl and methoxyl groups) at the pyrimidine unit of NHC ligands can significantly red-shift/blue-shift emission wavelength. DFT calculation results show that the LUMOs of these complexes mostly locate on the pyrimidine unit of NHC ligands (Figure 2), and it is well-known that electron-withdrawing groups can lower LUMO levels and electron-donating groups can raise LUMO levels, which should be the main reason of the emission wavelength red/blue-shifting for these complexes. Similar to previous our reports on Cu(I)-NHC complexes (Wang et al., 2018), the weaker electron-donating methyl group leads to a shorter emission wavelength in comparison with the stronger electron-donating methoxyl group. The reason should be that the imidazolylidene units of these NHC ligands have contributions to both HOMOs and LUMOs, because it means that electron-donating groups can raise HOMO levels as well as LUMO levels. For a similar reason, the emission wavelength of [Cu(Cl-Pmim)(POP)](PF_6_) is slightly longer than that of [Cu(F-Pmim)(POP)](PF_6_). The absolute photoluminescence quantum yields were measured to 7.4% for [Cu(Pmim)(POP)](PF_6_), 0.5% for [Cu(Cl-Pmim)(POP)](PF_6_), 0.9% for [Cu(F-Pmim)(POP)](PF_6_), 38.1% for [Cu(Me-Pmim)(POP)](PF_6_), and 20.1% for [Cu(MeO-Pmim)(POP)](PF_6_), respectively. It can be found that the electron-withdrawing/donating groups can obviously decrease/increase the emission efficiencies, which is similar to previous our reports on Cu(I)-NHC complexes (Wang et al., 2016, 2018).

**Figure 5:**
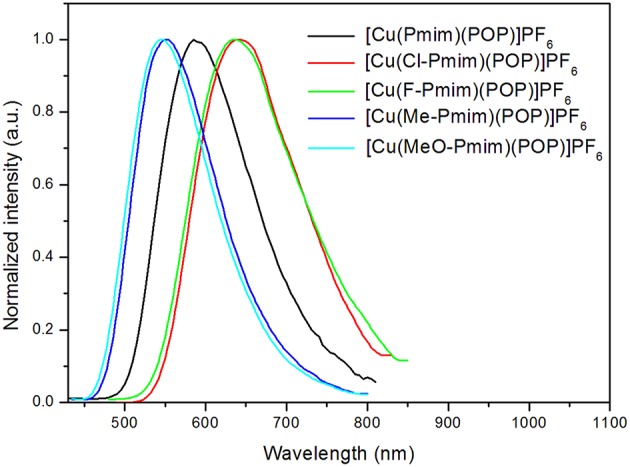
Photoluminescence spectra of the Cu(I)-NHC complexes in solid state at 50 K.

All of these complexes show microsecond-scale emission lifetimes, 6.3 μs for [Cu(Pmim)(POP)](PF_6_), 2.8 μs for [Cu(Cl-Pmim)(POP)](PF_6_), 4.5 μs for [Cu(F-Pmim)(POP)](PF_6_), 20.9 μs for [Cu(Me-Pmim)(POP)](PF_6_), and 9.6 μs for [Cu(MeO-Pmim)(POP)](PF_6_), respectively. To understand the emissive states of these complexes, photophysical properties under low-temperature condition were measured. The photoluminescence spectra of these complexes in solid state at 50 K are shown in Figure 5. The emission spectra measured at 50 K exhibit obvious red-shift (3–25 nm) compared to those acquired at room temperature (298 K). The emission lifetimes increased by a factor of about 3–10 when these complexes are cooled from 298 to 50 K (Table 2, Figure S3). Moreover, all of these complexes exhibit relatively small Δ*E*_ST_ from 0.15 to 0.36 eV (Table 2). The smaller Δ*E*_ST_, red-shift of photoluminescence spectra and increase of emission lifetimes upon decreasing measurement temperature implies that emissions of these complexes at room temperature are TADF.

**Table 2:** Emission data of the Cu(I)-NHC complexes in solid state at 298 and 50 K.

**Complex**	**298 K**			**50 K**		**S${}_{1}^{c}$ (eV)**	**T${}_{1}^{d}$ (eV)**	**Δ*E*_**ST**_ (eV)**
	**λ_**em**_ (nm)**	**τ**^a^** (μs)**	**Φ**^b^** (%)**	**λ_em_ (nm)**	**τ**^a^** (μs)**			
[Cu(Pmim)(POP)](PF_6_)	570	6.3	7.4	586	30.6	2.86	2.61	0.25
[Cu(Cl-Pmim)(POP)](PF_6_)	618	2.8	0.5	643	10.5	2.55	2.40	0.15
[Cu(F-Pmim)(POP)](PF_6_)	616	4.5	0.9	638	15.8	2.65	2.48	0.17
[Cu(Me-Pmim)(POP)](PF_6_)	530	20.9	38.1	552	72.8	3.11	2.75	0.36
[Cu(MeO-Pmim)(POP)](PF_6_)	542	9.6	20.1	545	100.3	3.12	2.78	0.34

a *Average lifetime which is calculated by the equation τ_ave_ = ∑A_i_τ_i_/∑A_i_ with A_i_ as the pre-exponential factor for the lifetime. The individual emission lifetimes are listed in Table S2*.

b *Absolute photoluminescence quantum yield with relative error = ±10%.cEstimated from the onset wavelength of emission spectra measured at 298 K. d Estimated from the onset wavelength of emission spectra measured at 50 K*.

## Conclusions

In summary, five new cationic mononuclear four-coordinate Cu(I) complexes consisted of the diphosphine ligand POP and the NHC ligands with imidazolylidene and pyrimidine units were successfully synthesized. The electron-donating groups at the pyrimidine unit of NHC ligands can significantly strengthen the Cu–N bonds and weaken the Cu–C bonds in these complexes, contrarily, the electron-withdrawing groups can lead to longer Cu–N bonds and shorter Cu–C bonds. Theoretical calculations show that these complexes have spatially separated HOMOs and LUMOs, and the lowest energy electronic transitions are mainly MLCT transition and LLCT transition. These complexes in solid state show wavelength-tunable emissions, the electron-donating/withdrawing groups at pyrimidine unit of the NHC ligands can significantly blue/red-shift emission wavelength and can obviously decrease/increase the emission efficiencies. The photophysical behaviors at 298 K and 50 K indicate that emissions of these Cu(I) complexes at room temperature are TADF.

## Author Contributions

ZW designed whole work. ZW and CX synthesized and characterized these compounds. ZW, XS, and BJ characterized the physical properties of complexes. All authors contributed to the general discussion.

### Conflict of Interest Statement

The authors declare that the research was conducted in the absence of any commercial or financial relationships that could be construed as a potential conflict of interest.

## References

[B1] Alkan-Zambada M. Keller S. Sessolo M. Ort,í E. Housecroft C. E. (2018). [Cu(P^∧^P)(N^∧^N)][PF_6_] compounds with bis(phosphane) and 6-alkoxy, 6-alkylthio, 6-phenyloxy and 6-phenylthio-substituted 2,2′-bipyridine ligands for light-emitting electrochemical cells. J. Mater. Chem. C 6, 8460–8471. 10.1039/C8TC02882F

[B2] Armaroli N. Accorsi G. Holler M. Nierengarten J.-F. Wwnh R. T. Welter R. (2006). Highly luminescent Cu^I^ complexes for light-emitting electrochemical cells. Adv. Mater. 18, 1313–1316. 10.1002/adma.200502365

[B3] Brown C. M. Carta V. Wolf M. O. (2018). Thermochromic solid-state emission of dipyridyl sulfoxide Cu(I) complexes. Chem. Mater. 30, 5786–5795. 10.1021/acs.chemmater.8b02821

[B4] Brunner F. Babaei A. Pertegás A. Ort,í E. Housecroft C. E. (2019). Phosphane tuning in heteroleptic [Cu(N^∧^N)(P^∧^P)]^+^ complexes for light-emitting electrochemical cells. Dalton Trans. 48, 446–460. 10.1039/C8DT03827A 30452034

[B5] Brunner F. Graber S. Baumgartner Y. Häussinger D. Prescimone A. Constable E. C. (2017). The effects of introducing sterically demanding aryl substituents in [Cu(N^∧^N)(P^∧^P)]^+^ complexes. Dalton Trans. 46, 6379–6391. 10.1039/C7DT00782E 28466927

[B6] Cheng G. So G. K.-M. To W.-P. Chen Y. Che C.-M.. (2015). Luminescent zinc(II) and copper(I) complexes for high-performance solution-processed monochromic and white organic light-emitting devices. Chem. Sci. 6, 4623–4635. 10.1039/C4SC03161J 29142704 PMC5667404

[B7] Cuttell D. G. Kuang S.-M. Fanwick P. E. McMillin D. R. Walton R. A. (2002). Simple Cu(I) complexes with unprecedented excited-state lifetimes. J. Am. Chem. Soc. 124, 6–7. 10.1021/ja012247h 11772046

[B8] Elie M. Sguerra F. Linares M. Hamel M. Costa R. D. Gaillard S.. (2016). Designing NHC–copper(I) dipyridylamine complexes for blue light-emitting electrochemical cells. ACS. Appl. Mater. Interfaces 8, 14678–14691. 10.1021/acsami.6b04647 27224961

[B9] Felder D. Nierengarten J.-F. Barigelletti F. Ventura B. Armaroli N. (2001). J. Am. Chem. Soc. 123, 6291–6299. 10.1021/ja0043439 11427053

[B10] Gneuß T. Leitl M. J. Finger L. H. Yersin H. Sundermeyer J. (2015). A new class of deep-blue emitting Cu(I) compounds–effects of counter ions on the emission behavior. Dalton Trans. 44, 20045–20055. 10.1039/c5dt03065j 26525145

[B11] Hamze R. Jazzar R. Soleilhavoup M. Djurovich P. I. Bertrand G. Thompson M. E. (2017). Phosphorescent 2-, 3- and 4-coordinate cyclic (alkyl)(amino)carbene (CAAC) Cu(I) complexes. Chem. Commun. 53, 9008–9011. 10.1039/C7CC02638B 28745735

[B12] He L.-H. Luo Y.-S. Chen J.-L. Ho C.-L. Wang J.-Y. Wong W.-Y.. (2017). Luminescent three- and four-coordinate dinuclear copper(I) complexes triply bridged by bis(diphenylphosphino)methane and functionalized 3-(2′-pyridyl)-1,2,4-triazole ligands. Inorg. Chem. 56, 10311–10324. 10.1021/acs.inorgchem.7b01159 28825489

[B13] Hofbeck T. Monkowius U. Yersin H. (2015). Highly efficient luminescence of Cu(I) compounds: thermally activated delayed fluorescence combined with short-lived phosphorescence. J. Am. Chem. Soc. 137, 399–404. 10.1021/ja5109672 25486064

[B14] Huang C.-H. Wen M. Wang C.-Y. Huang X.-H. Li H.-H.. (2017). A series of pure-blue-light emitting Cu(I) complexes with thermally activated delayed fluorescence: structural, photophysical, and computational studies. Dalton Trans. 46, 1413–1419. 10.1039/C6DT03965K 28045159

[B15] Jia J.-H. Chen X.-L. Liao J.-Z. Liang D. Yang M.-X. Yu R.. (2019). Highly luminescent copper(I) halide complexes chelated with a tetradentate ligand (PNNP): synthesis, structure, photophysical properties and theoretical studies. Dalton Trans. 48, 1418–1426. 10.1039/C8DT03452D 30628618

[B16] Kalsani V. Schmittel M. (2006). Novel phenanthroline ligands and their kinetically locked copper(I) complexes with unexpected photophysical properties. Inorg. Chem. 45, 2061–2067. 10.1021/ic051828v 16499367

[B17] Keller S. Prescimone A. Bolink H. Ort,í E. Housecroft C. E.. (2018). Luminescent copper(I) complexes with bisphosphane and halogen-substituted 2,2′-bipyridine ligands. Dalton Trans. 47, 14263–14276. 10.1039/C8DT01338A 29790540

[B18] Kobayashi A. Hasegawa T. Yoshida M. Kato M. (2016). Environmentally friendly mechanochemical syntheses and conversions of highly luminescent Cu(I) dinuclear complexes. Inorg. Chem. 55, 1978–1985. 10.1021/acs.inorgchem.5b02160 26866384

[B19] Kovalevsky A. Y. Gembicky M. Novozhilova I. V. Coppens P. (2003). Solid-state structure dependence of the molecular distortion and spectroscopic properties of the Cu(I) bis(2,9-dimethyl-1,10-phenanthroline) ion. Inorg. Chem. 42, 8794–8802. 10.1021/ic0348805 14686859

[B20] Krylova V. A. Djurovich P. I. Aronson J. W. Haiges R. Whited M. T. Thompson M. E. (2012). Dinuclear coinage-metal complexes of bis(NHC) ligands: structural features and dynamic behavior of a Cu–Cu complex. Organometallics 31, 7983–7901. 10.1021/om300544g

[B21] Kuang S.-M. Cuttell D. G. McMillin D. R. Fanwick P. E. Walton R. A. (2002). Synthesis and structural characterization of Cu(I) and Ni(II) complexes that contain the bis[2-(diphenylphosphino)phenyl]ether ligand. novel emission properties for the Cu(I) species. Inorg. Chem. 41, 3313–3322. 10.1021/ic0201809 12055011

[B22] Leitl M. J. Krylova V. A. Djurovich P. I. Thompson M. E. Yersin H. (2014). Phosphorescence versus thermally activated delayed fluorescence. Controlling singlet–triplet splitting in brightly emitting and sublimable Cu(I) compounds. J. Am. Chem. Soc. 136, 16032–16038. 10.1021/ja508155x 25260042

[B23] Leydet Y. Bassani D. M. Jonusauskas G. McClenaghan N. D. (2007). Equilibration between three different excited states in a bichromophoric copper(I) polypyridine complex. J. Am. Chem. Soc. 129, 8688–8689. 10.1021/ja072335n 17585767

[B24] Liang D. Chen X.-L. Liao J.-Z. Hu J.-Y. Jia J.-H. Lu C.-Z. (2016). Highly efficient cuprous complexes with thermally activated delayed fluorescence for solution-processed organic light-emitting devices. Inorg. Chem. 55, 7467–7475. 10.1021/acs.inorgchem.6b00763 27404980

[B25] Lin L. Chen D.-H. Yu R. Chen X.-L. Lu C.-Z. (2017). Photo- and electro-luminescence of three TADF binuclear Cu(I) complexes with functional tetraimine ligands. J. Mater. Chem. C 5, 4495–4504. 10.1039/C7TC00443E

[B26] Liu L.-P. Li Q. Liu L. Li G. H. Li F.-B. Wong W.-Y.. (2018). Near-saturated red emitters: four-coordinate copper(I) halide complexes containing 8-(diphenylphosphino)quinoline and 1-(diphenylphosphino)naphthalene ligands. Dalton Trans. 47, 9294–9302. 10.1039/C7DT04528J 29878015

[B27] Liu S. Xu S. Wang J. Zhao F. Xia H. Wang Y. (2017). Four-coordinate N-heterocyclic carbene (NHC) copper(I) complexes with brightly luminescence properties. J. Coord. Chem. 70, 584–599. 10.1080/00958972.2016.1278075

[B28] Lu T. Wang J.-Y. Shi L.-X. Chen Z.-N. Chen X.-T. Xue Z.-L. (2018). Synthesis, structures and luminescence properties of amine-bis(N-heterocyclic carbene) copper(I) and silver(I) complexes. Dalton Trans. 47, 6742–6753. 10.1039/C8DT00599K 29713716

[B29] Marion R. Sguerra F. Linares M. Hamel M. Gaillard S.. (2014). NHC copper(I) complexes bearing dipyridylamine ligands: synthesis, structural, and photoluminescent studies. Inorg. Chem. 53, 9181–9191. 10.1021/ic501230m 25134011

[B30] Mohankumar M. Holler M. Nierengarten J.-F. Sauvage J.-P. Delavaux-Nicot B. Armaroli N.. (2018). Heteroleptic copper(I) pseudorotaxanes incorporating macrocyclic phenanthroline ligands of different sizes. J. Am. Chem. Soc. 140, 2336–2347. 10.1021/jacs.7b12671 29298047

[B31] Nishikawa M. Sano T. Washimi M. Takao K. Tsubomura T. (2016). Emission properties and Cu(I)–Cu(I) interaction in 2-coordinate dicopper(I)-bis(N-heterocyclic)carbene complexes. Dalton Trans. 45,12127–12136. 10.1039/C6DT01239F 27399152

[B32] Osawa M. Hoshino M. Hashimoto M. Kawata I. Igawa S. Yashima M. (2015). Application of three-coordinate copper(I) complexes with halide ligands in organic light-emitting diodes that exhibit delayed fluorescence. Dalton Trans. 43, 8369–8378. 10.1039/C4DT02853H 25470470

[B33] Scaltrito D. V. Thompson D. W. O'Callaghan J. A. Meyer G. J. (2000). Coord. Chem. Rev. 208, 243–1016.

[B34] Schinabeck A. Rau N. Klein M. Sundermeyer J. Yersin H. (2018). Deep blue emitting Cu(I) tripod complexes. Design of high quantum yield materials showing TADF-assisted phosphorescence. Dalton Trans. 47, 17067–17076. 10.1039/C8DT04093A 30465052

[B35] Shi Y.-Z. Wang K. Li X. Dai G.-L. Liu W. Zheng C.-J.. (2018). Intermolecular charge-transfer transition emitter showing thermally activated delayed fluorescence for efficient non-doped OLEDs. Angew. Chem. Int. Ed. 57, 9480–9484. 10.1002/anie.201804483 29863299

[B36] Simon J. A. Palke W. E. Ford P. C. (1996). Photophysical and *ab initio* studies of mononuclear copper(I) complexes. Inorg. Chem. 35, 6413–6421. 10.1021/ic960367y 11666788

[B37] Su Z. C. Zheng C. C. Cheng G. Che C.-M. Xu S. J. (2017). Triplet harvesting in luminescent Cu(I) complexes by the thermally activated luminescence transition mechanism: impact of the molecular structure. J. Mater. Chem. C 5, 4488–4494. 10.1039/C7TC00773F

[B38] Volz D. Wallesch M. Grage S. L. Heske C. Weinhardt L. Baumann T. Bräse, S.. (2014). Labile or stable: can homoleptic and heteroleptic pyrPHOS–copper complexes be processed from solution? Inorg. Chem. 53, 7837–7847. 10.1021/ic500135m 25028770

[B39] Wang J. Liu S. Xu S. Zhao F. Xia H. Wang Y. (2017). Four-coordinated copper(I) complexes containing variably substituted N-heterocyclic carbenes (NHCs): Synthesis, photophysical properties and theoretical investigation. J. Organomet. Chem. 846, 351–359. 10.1016/j.jorganchem.2017.07.016

[B40] Wang K. Zheng C.-J. Liu W. Liang K. Lee C.-S. Zhang X.-H.. (2017). Avoiding energy loss on TADF emitters: controlling the dual conformations of D–A structure molecules based on the pseudoplanar segments. Adv. Mater. 29:1701476. 10.1002/adma.201701476 29116652

[B41] Wang L. Liu N. Dai B. (2015). Metal-free site-selective C–N bond-forming reaction of polyhalogenated pyridines and pyrimidines. RSC Adv. 5, 82097–82111. 10.1039/C5RA18653F

[B42] Wang Z. Cai J. Zhang M. Zheng C. Ji B. (2019). A novel yellow thermally activated delayed fluorescence emitter for highly efficient organic light-emitting diodes. Acta Chim. Sinica 77:263–268. 10.6023/A18100437

[B43] Wang Z. Sun X. Fu W. Xu C. Ji B. (2018). Four-coordinate Cu(I) complexes supported by N-heterocyclic carbine ligands bearing electron-donating/withdrawing groups: synthesis, structures and photophysical properties. J. Lumin. 204, 618–625. 10.1016/j.jlumin.2018.08.064

[B44] Wang Z. Zheng C. Wang W. Xu C. Ji B. Zhang X. (2016). Synthesis, structure, and photophysical properties of two four-coordinate Cu^I^-NHC complexes with efficient delayed fluorescence. Inorg. Chem. 55, 2157–2164. 10.1021/acs.inorgchem.5b02546 26907724

[B45] Xu S. Wang J. Liu S. Zhao F. Xia H. Wang Y. (2018). Synthesis, photophysical properties, and computational studies of four-coordinate copper(I) complexes based on benzimidazolylidene N-heterocyclic carbene (NHC) ligands bearing aryl substituents. J. Mol. Struct. 1153, 12–19. 10.1016/j.molstruc.2017.09.119

[B46] Yang Z. Mao Z. Xie Z. Zhang Y. Liu S. Zhao J.. (2017). Recent advances in organic thermally activated delayed fluorescence materials. Chem. Soc. Rev. 46, 915–1016. 10.1039/C6CS00368K 28117864

[B47] Zhang F. Guan Y. Chen X. Wang S Liang D. Feng Y.. (2017). Syntheses, photoluminescence, and electroluminescence of a series of sublimable bipolar cationic cuprous complexes with thermally activated delayed fluorescence. Inorg. Chem. 56, 3742–3753. 10.1021/acs.inorgchem.6b01847 28304161

[B48] Zhang Q. Zhou Q. Cheng Y. Wang L. Ma D. Jing X. (2004). Highly efficient green phosphorescent organic light-emitting diodes based on Cu^I^ complexes. Adv. Mater. 16, 432–436. 10.1002/adma.200306414

