# Supplementary material for: Synthesis, Structures, and Photophysical Properties of Novel Four-Coordinate Cu(I) Complexes Supported by Chelating N-Heterocyclic Carbene Ligands
Source: Front Chem. 2019 Jun 5;7:422. doi: 10.3389/fchem.2019.00422 (PMC6561309; doi:10.3389/fchem.2019.00422)
Supplement: Supplementary file 1 [file Table_1.docx]

Supplementary Material


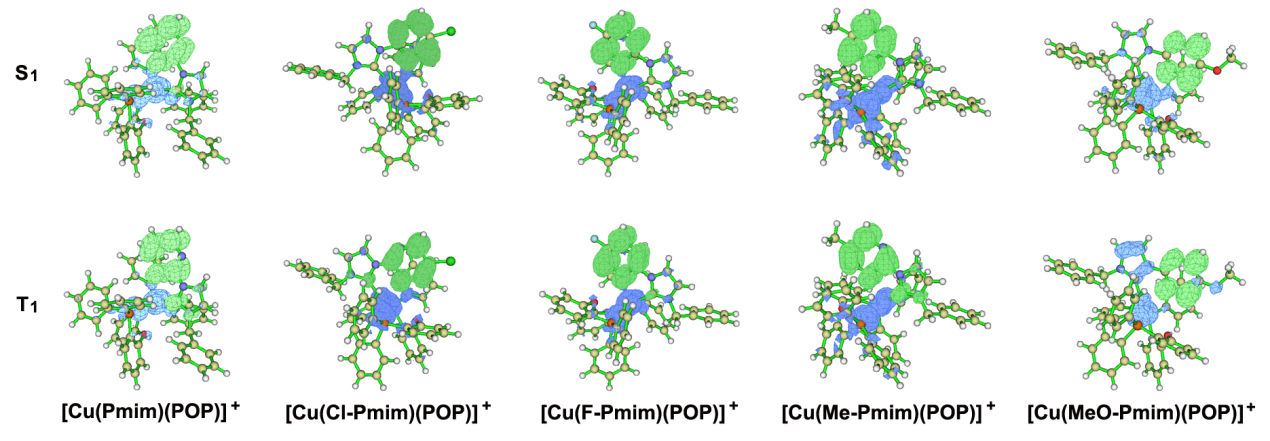


**Figure S1** Calculated hole and electron distributions of [Cu(Pmim)(POP)](PF_6_), [Cu(Cl-Pmim)(POP)](PF_6_), [Cu(F-Pmim)(POP)](PF_6_), [Cu(Me-Pmim)(POP)](PF_6_) and [Cu(MeO-Pmim)(POP)](PF_6_) at S_1_ and T_1_ states.

**

**

**Figure S2** Absorption spectra of the free ligand POP and the NHC ligand precursors **6**, **7**, **8**, **9** and **10** in CH_2_Cl_2_ solution at room temperature.





**(A)**







**(B) (C)**







**(D) (E)**

**Figure S3** Emission decay behaviors of the Cu(I)-NHC complexes in solid state at 298 K and 50 K, **(A)** [Cu(Pmim)(POP)](PF_6_), **(B)** [Cu(Cl-Pmim)(POP)](PF_6_), **(C)** [Cu(F-Pmim)(POP)](PF_6_), **(D)** [Cu(Me-Pmim)(POP)](PF_6_) and **(E)** [Cu(MeO-Pmim)(POP)](PF_6_).

**Table S1** Calculated lowest energy transitions for the Cu(I)-NHC complexes.

| complexes | states | energy (eV) | *f* | major contribution |
| --- | --- | --- | --- | --- |
| [Cu(Pmim)(POP)](PF_6_) | S_1_ | 3.2888 | 0.0333 | HOMO→LUMO (99%) |
|  | T_1_ | 3.1112 | 0 | HOMO→LUMO (78%) |
|  |  |  |  | HOMO→LUMO+1 (19%) |
|  | T_2_ | 3.2226 | 0 | HOMO-1→LUMO (47%) |
|  |  |  |  | HOMO-1→LUMO+1 (26%) |
|  |  |  |  | HOMO-3→LUMO (13%) |
| [Cu(Cl-Pmim)(POP)](PF_6_) | S_1_ | 3.1121 | 0.0212 | HOMO→LUMO (99%) |
|  | T_1_ | 2.9894 | 0 | HOMO→LUMO (87%) |
|  |  |  |  | HOMO→LUMO+1 (13%) |
|  | T_2_ | 3.1512 | 0 | HOMO-1→LUMO (50%) |
|  |  |  |  | HOMO-1→LUMO+1 (24%) |
|  |  |  |  | HOMO-3→LUMO (21%) |
| [Cu(F-Pmim)(POP)](PF_6_) | S_1_ | 3.0973 | 0.0176 | HOMO→LUMO (99%) |
|  | T_1_ | 2.9940 | 0 | HOMO→LUMO (94%) |
|  | T_2_ | 3.1780 | 0 | HOMO-1→LUMO (53%) |
|  |  |  |  | HOMO-1→LUMO+1 (12%) |
|  |  |  |  | HOMO-3→LUMO (23%) |
| [Cu(Me-Pmim)(POP)](PF_6_) | S_1_ | 3.3060 | 0.0253 | HOMO→LUMO (99%) |
|  | T_1_ | 3.1491 | 0 | HOMO→LUMO (72%) |
|  |  |  |  | HOMO→LUMO+1 (18%) |
|  | T_2_ | 3.2779 | 0 | HOMO-1→LUMO (44%) |
|  |  |  |  | HOMO-1→LUMO+1 (22%) |
|  |  |  |  | HOMO-2→LUMO (16%) |
|  |  |  |  | HOMO→LUMO (12%) |
| [Cu(MeO-Pmim)(POP)](PF_6_) | S_1_ | 3.2383 | 0.0132 | HOMO→LUMO (99%) |
|  | T_1_ | 3.1119 | 0 | HOMO→LUMO (78%) |
|  | T_2_ | 3.2732 | 0 | HOMO-1→LUMO (26%) |
|  |  |  |  | HOMO-2→LUMO (8%) |
|  |  |  |  | HOMO-4→LUMO (18%) |
|  |  |  |  | HOMO-5→LUMO (15%) |
|  |  |  |  | HOMO→LUMO+1 (8%) |

**Table S2** The individual emission lifetimes of the Cu(I)-NHC complexes as crystal powder at 298 K and 50 K.

| Complexes | Emission lifetimes (μs) | |
| --- | --- | --- |
|  | 298 K | 50 K |
| [Cu(Pmim)(POP)](PF_6_) | 4.68 (69.13%)  25.83 (30.87%) | 30.59 |
| [Cu(Cl-Pmim)(POP)](PF_6_) | 1.02 (12.18%)  4.64 (14.91%)  73.58 (72.91%) | 7.20 (38.52%)  13.43 (61.48%) |
| [Cu(F-Pmim)(POP)](PF_6_) | 1.04 (14.06%)  3.81 (26.62%)  36.20 (59.33%) | 1.75 (19.16%)  13.79 (80.84%) |
| [Cu(Me-Pmim)(POP)](PF_6_) | 20.99 | 42.38 (41.04%)  145.2 (58.96%) |
| [Cu(MeO-Pmim)(POP)](PF_6_) | 5.11 (22.59%)  13.15 (77.41%) | 69.60 (29.40%)  122.8 (70.60%) |
